# Genetic analysis for mucinous ovarian carcinoma with infiltrative and expansile invasion and mucinous borderline tumor: a retrospective analysis

**DOI:** 10.1186/s13000-023-01340-w

**Published:** 2023-04-20

**Authors:** Taira Hada, Morikazu Miyamoto, Yuka Ohtsuka, Jin Suminokura, Tsubasa Ito, Naohisa Kishimoto, Soko Nishitani, Minori Takada, Akari Imauji, Risa Tanabe, Masashi Takano

**Affiliations:** Department of Obstetrics and Gynecology, National Defense Medical College Hospital, 3-2, Namiki, Tokorozawa, Saitama 359-8513 Japan

**Keywords:** Ovarian mucinous carcinoma, Infiltrative invasion, Expansile invasion, Mucinous borderline tumor, Genetic examination, Tumor mutation burden

## Abstract

**Background:**

Mucinous carcinoma (MC) is a histological subtype of ovarian cancer that has a worse prognosis at advanced stages than the most prevalent histological subtype, high-grade serous carcinomas. Invasive patterns have been recognized as prognostic factors for MCs. MCs with infiltrative invasion were more aggressive than those with expansile invasion. MC with an expansile pattern exhibited behavior similar to mucinous borderline tumors (MBT). However, genomic analysis of invasive patterns is insufficient. This study aimed to compare genetic information between groups with MC and infiltrative invasion (Group A) and those with MC with expansile invasion or MBT (Group B).

**Methods:**

Ten cases each of MC with infiltrative invasion, MC with expansile invasion, and MBT between 2005 and 2020 were identified. Deoxyribonucleic acid (DNA) extraction from formalin-fixed paraffin-embedded tissues was performed, and cases with DNA fragmentation or the possibility of DNA fragmentation were excluded. Mutant base candidates and tumor mutation burden (TMB) values (mutations/megabase) were calculated.

**Results:**

After assessing the quality of purified DNA, seven cases of MC with infiltrative invasion, five cases of MC with expansile invasion, and three cases of MBT were included. More patients in group A experienced recurrence or progression (*p* < 0.01) and died of disease (*p* = 0.03). Moreover, the TMB value was statistically higher in group A than in group B (*p* = 0.049). There were no statistical differences in the incidence of the mutations of KRAS, TP53, and CREBBP. KRAS, TP53, and CREBBP mutations were discovered in 8/15 (53.3%), 6/15 (40.0%), and 5/15 (33.3%) cases, respectively.

**Conclusions:**

Genetic analysis revealed that Group A had higher TMB than Group B. Therefore, this result might be useful for future treatment.

## Background

Epithelial ovarian carcinoma (EOC) is one of the most lethal gynecological diseases [1, 2]. The standard treatment of EOCs is debulking surgery followed by adjuvant platinum-based chemotherapy [3]. Poly ADP-ribose inhibitors (PARPis) have recently been used as maintenance therapy options for EOCs, and breast cancer susceptibility gene (BRCA) and homologous recombination status are important biomarkers for their use [3]. Thus, genetic information has become a fundamental factor in selecting the treatment of EOCs.

According to the 2020 World Health Organization (WHO) classification, EOCs are morphologically classified into several histological subtypes. Among them, mucinous carcinoma (MC), has an incidence ranging from 3 to 11% [4, 5]. The majority of cases of MC are diagnosed at earlier International Federation of Obstetrics and Gynecology (FIGO) stages and have better prognoses than other histological subtypes. However, MC discovered at advanced FIGO stages have worse prognoses than the most prevalent histological subtype, high-grade serous carcinoma [5, 6].

Based on histological evaluation of invasive patterns, MCs are classified into two types: infiltrative invasion and expansile invasion [7]. Compared to MC with expansile invasion, MC with infiltrative invasion is typically discovered at more advanced FIGO stages and is related to a worse prognosis. Therefore, it is considered the more aggressive type [8–11]. In contrast, MC with expansile invasion has a clinical behavior similar to that of mucinous borderline tumors (MBTs) [12]. Thus, invasive patterns are important factors to consider in the clinical management of MCs, and the European Society for Medical Oncology and European Society of Gynecological Oncology guidelines recommend different treatments according to invasive patterns [13].

According to previous literature, MC is associated with genetic alterations such as KRAS mutations, tumor suppressor protein p53 (TP53) mutations, and human epidermal growth factor receptor 2 (HER2) amplifications, but an effective treatment for MCs has not been established in spite of these identified gene alterations [14]. Furthermore, PARPi was assumed to have low efficacy for MCs because many cases of MCs lack BRCA mutations or defects in homologous recombination [15, 16]. Therefore, we considered that new genomic information would be useful in developing effective treatments for MC.

Therefore, this study aimed to evaluate the genetic information of MC with infiltrative invasion, MC with expansile invasion, and MBT.

## Methods

Ten patients with MC with infiltrative invasion, 10 with MC with expansile invasion, and 10 with MBT who underwent primary surgery between 2005 and 2020 at National Defense Medical College were included. Cases with a prior history of chemotherapy and with destructive deoxyribonucleic acid (DNA) fragmentation or the possibility of DNA fragmentation were excluded.

The pathological diagnosis was conducted based on the 2020 WHO classification by experienced gynecological pathologist at National Defense Medical College [4]. In case that even a little area of the component of MC with infiltrative invasion was observed, the case was diagnosed as MC with infiltrative invasion. Also, in case that even a little area of the component of MC with expansile invasion and no component of MC with infiltrative invasion was observed, the case was diagnosed as MC with expansile invasion. The cases which contained the component of MBT only were diagnosed as MBT.

Staging was performed using the 2014 FIGO criteria [17]. Data on residual tumors after primary surgery were obtained from the surgical records of the primary surgery. All the cases underwent postoperatively gastroscopy and colonofiberscopy, cervical cytology, abdominopelvic contrast-enhanced computed tomography examination, Magnetic Resonance Imaging. We used surgical specimen and performed not only hematoxylin and eosin stain but also immunohistochemistry for cytokeratin 7, cytokeratin 20, caudal-related homeobox transcription factor 2, and paired box 8 to exclude the metastatic ovarian carcinoma originating from other sites like the gastrointestinal tract, pancreaticobiliary tract, and endocervical mucinous adenocarcinomas. In addition, when swelling of the appendix was observed intraoperatively, appendectomy was performed by a digestive surgeon to exclude appendiceal cancer.

DNA was extracted from formalin-fixed paraffin-embedded (FFPE) tissues using NucleoSpin DNA FFPE XS (Macherey Nagel, Düren, Germany) according to the manufacturer’s instructions (Takara Bio, Shiga, Japan) [18]. The areas which contained the purely component of MC with infiltrative invasion, MC with expansile invasion, and MBT, respectively, were selected by gynecological pathologist for extracting DNA. The quality of the purified DNA was assessed using the Qubit dsDNA HS Assay Kit (Thermo Fisher Scientific, Inc., Massachusetts, USA), TaqMan Universal PCR Master Mix (Thermo Fisher Scientific), and TaqMan RNase P Detection Reagent Kit (Thermo Fisher Scientific) according to the manufacturer’s protocol (Takara Bio) [18].

Mutant base candidates and tumor mutation burden (TMB) values (Mutations/Megabase [Mut/Mb]) were detected by mapping the sequence results obtained using sequence analysis on the Torrent Server according to the Ion Reporter 5.12, according to the Oncomine™ Tumor Mutation Load Assay USER GUIDE (Rev. C.0) [18]. Briefly, the average base coverage depth to detect 5% or greater of the mutant base candidates was 1100 or greater, and the TMB values were calculated to be 300 or greater. The detection of mutant base candidates may result in more false negatives when the average depth is less than 1,100. Formalin-fixation could induce C:G > T:A transitions, and that would lead to an artificial increase in the number of mutations and result in an inaccurate TMB estimation. Potential deamination artifacts were defined as the number of C:G *>* T:A mutations with an allelic frequency *<* 15% in coding regions, and statistical analyses were performed using Oncomine™ Tumor Mutation Load Assay USER GUIDE (Rev. C.0).

Statistical analysis was performed using the JMP Pro 14 software (SAS Institute Inc., Cary, NC, USA). The chi-square test, Fisher’s exact test, Mann-Whitney U test, and Wilcoxon test were used to evaluate the statistical significance of the clinical and genetic factors. Group A was defined as patients with MC with infiltrative invasion and Group B was defined as patients with MC with expansile invasion and MBT. The level of statistical significance was set at *p* < 0.05.

## Results

**Fig. 1:**
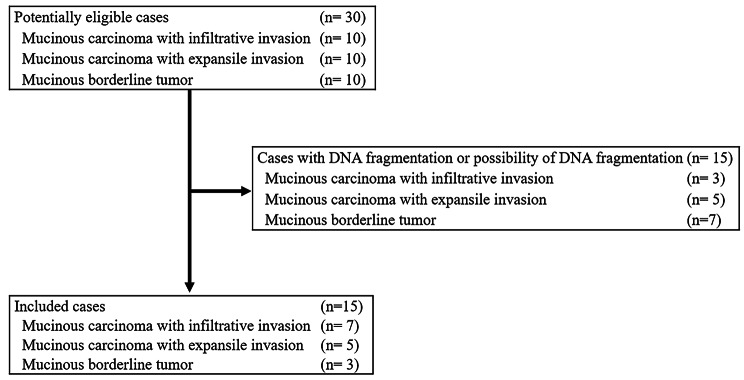
The diagram of this study. Ten cases each of mucinous carcinoma (MC) with infiltrative invasion, MC with expansile invasion, and mucinous borderline tumor (MBT) were initially identified. After assessing the quality of the purified deoxyribonucleic acid (DNA), cases of DNA fragmentation or the possibility of DNA fragmentation were excluded. Finally, 15 cases were included: seven cases of MC with infiltrative invasion, five cases of MC with expansile invasion, and three cases of MBT.

After the analysis to check the quality of DNA, seven cases of MC with infiltrative invasion, five of MC with expansile invasion, and three of MBT were included (Fig. 1). There were no cases which contained purely infiltrative pattern, expansile pattern, or borderline tumor.

Comparisons of the characteristics between Group A and Group B are shown in Table 1. There were no statistical differences in age, FIGO stage, residual tumor after primary surgery, peritoneal cytology, and endometriosis between the two groups. More patients in Group A experienced recurrence or progression (*p* < 0.01) and died of the disease (*p* = 0.03) than in Group B.

**Table 1:** Characteristics of patients with mucinous carcinoma with infiltrative invasion (Group A) and patients with mucinous carcinoma with expansile invasion and mucinous borderline tumor (Group B)

	Group A		Group B		*p*-value
Variables	(n = 7)		(n = 8)		
Age (average ± SD)	49.3 ± 16.9		59.0 ± 21.9		0.22
FIGO stage (%)					0.99
I	5	(71.4)	6	(75.0)	
II	0	(0.0)	1	(12.5)	
III	1	(14.3)	0	(0.0)	
IV	1	(14.3)	1	(12.5)	
Residual tumor after primary surgery (%)					0.57
Yes	2	(28.6)	1	(12.5)	
No	5	(71.4)	7	(87.5)	
Peritoneal cytology (%)					0.19
Positive	4	(57.1)	1	(12.5)	
Negative	3	(42.9)	7	(87.5)	
Endometriosis (%)					0.19
Yes	4	(57.1)	1	(12.5)	
No	3	(42.9)	7	(87.5)	
Recurrence (%)					< 0.01
Yes	5	(71.4)	0	(0.0)	
No	2	(28.6)	8	(100.0)	
Died of disease (%)					0.03
Yes	4	(57.1)	0	(0.0)	
No	3	(42.9)	8	(100.0)	

Abbreviations: SD, standard deviation; FIGO, International Federation of Obstetrics and Gynecology

**Fig. 2:**
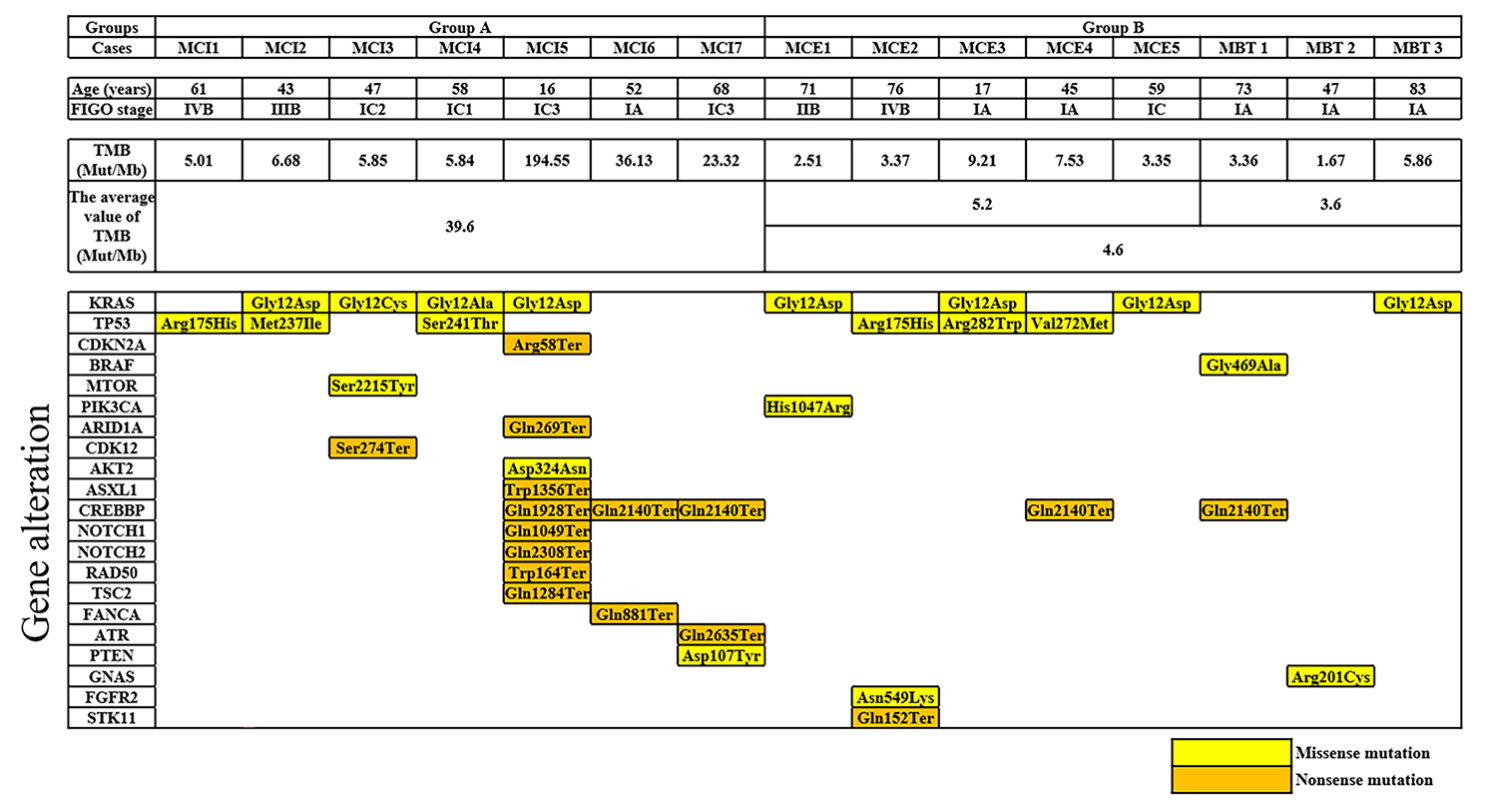
The value of tumor mutation burden and alteration of bases according to histological subtypes. The TMB value of group A was significantly higher than that of group B. Alterations in KRAS, tumor suppressor protein p53 (TP53), and cyclic adenosine monophosphate response element binding protein binding protein (CREBBP) were recognized in eight, six, and five cases, respectively. Other bases with alterations listed included one case each Other abbreviations MCI, mucinous carcinoma with infiltrative invasion. MCE, mucinous carcinoma with expansile invasion. MBT, mucinous borderline tumor; FIGO, International Federation of Obstetrics and Gynecology; TMB, tumor mutation burden; Mut, mutations; Mb, megabase; TP53, tumor suppressor protein p53; CDKN2A, cyclin-dependent kinase inhibitor 2 A; BRAF, v-raf murine sarcoma viral oncogene homolog B1; MTOR, Mammalian target of rapamycin; PIK3CA, phosphatidylinositol-4,5-bisphosphate 3kinase catalytic subunit alpha; ARID1A, the AT-rich interactive domain 1 A; CDK12, Cyclin-dependent kinase 12; CREBBP, cyclic adenosine monophosphate response element binding protein binding protein; NOTCH, Neurogenic locus notch homolog protein; TSC2, Tuberous Sclerosis Complex 2; FANCA, Fanconi anaemia, complementation group A; ATR, Ataxia-Telangiectasia-mutated- and Rad3-related; PTEN, phosphatase and tensin homolog; GNAS, Galpha encoding guanine nucleotide binding protein, alpha-stimulating; FGFR2, Fibroblast growth factor receptor 2; STK11, Serine/threonine kinase 11

The TMB value (Mut/Mb), and the mutation of bases according to MC with infiltrative invasion, MC with expansile invasion, and MBT are shown in Fig. 2. Potential deamination artifacts which affected the TMB value were not observed in all cases. The average TMB values in Group A and Group B were 39.6 Mut/Mb and 4.6 Mut/Mb, respectively, and the TMB values in Group A was statistically higher compared with Group B (*p* = 0.049). Mutations in KRAS, TP53, and cyclic adenosine monophosphate response element-binding protein-binding protein (CREBBP) were recognized in 8/15 (53.3%), 6/15 (40.0%), and 5/15 (33.3%) cases, respectively. The other mutational bases listed included one case each. There were no statistically significant differences in gene mutation patterns between the two groups.

## Discussion

In our study, mutations in KRAS, TP53, and CREBBP were identified. The TMB value of MC with infiltrative invasion was higher than that of MC with expansile invasion and MBT.

The clinical features of MC, such as FIGO stage, response rate to adjuvant chemotherapy, recurrence rate, and prognosis, may vary according to the invasive pattern [8–11]. MC with infiltrative invasion has a worse prognosis than MC with expansile invasion and high-grade serous carcinoma [11, 17]. Thus, MC with infiltrative invasion is an aggressive subtype. Hence, MC with expansile invasion has a better prognosis than MC with invasive invasion and high-grade serous carcinoma [19]. Furthermore, there were no statistically significant differences in the recurrence rate and prognosis of MC with expansile invasion and MBT [12]. Therefore, because MC with expansile invasion is a less aggressive histological subtype similar to MBT, the two histological subtypes were combined into one group in our study.

Histologically, EOCs are classified into type I and type II based on distinctive morphological and molecular genetic features, and MC are classified as type I [20, 21]. The incidence of mutations in KRAS, TP53, and BRAF ranges from 13.0 to 70.0%, from 52.0 to 64.0%, and from 5.4 to 22.6% of cases of MC, respectively [22–28]. In this study, although the frequencies of these mutations were different, several mutations were found, regardless of the group.

The amplification of HER2 occurs in 18.2–45.5% of MC cases, and a recent study proposed trastuzumab therapy as a treatment option for MC with HER2 amplification [29–31]. However, the amplification of HER2 was not observed in this study, possibly due to the small number of cases. Thus, further studies including more cases are needed, especially as HER2 inhibitor is considered a new treatment.

TMB is the number of somatic nonsynonymous mutations per coding area of the tumor genome. It is considered an important biomarker for predicting the response rate to immune checkpoint inhibitors (ICIs) [32, 33]. Compared to lower TMB values, a high TMB value is related to a greater response to ICIs [34, 35]. According to the previous literature, the average TMB value of EOCs is 5.3 Mut/Mb, and patients with EOCs may not benefit from ICIs as much as other types of carcinomas, such as lung carcinomas or melanoma [36, 37]. These studies included several cases of high-grade serous carcinomas. In contrast, a recent study reported that the average TMB value of MC was higher than that of high-grade serous carcinoma and low-grade serous carcinoma [38]. In this study, among MCs, the average TMB value of MC with an infiltrative pattern was higher than that of MC with expansile invasion and MBT. Our results indicate that ICIs might be a useful therapeutic option for MC with infiltrative invasion.

This study had some limitations. First, our study included small sample size, especially only 3 cases with MBT, from a single institution and retrospective nature. Second, our study did not perform individual cases with any combinations of mucinous ovarian carcinomas with infiltrative invasion, mucinous ovarian carcinomas with expansile invasion, and mucinous borderline tumors. Therefore, We could not yield answers regarding progression. Third, immunohistochemistry for mismatch repair proteins, germline genetic testing and investigating the status of major histocompatibility complex/human leukocyte antigen were not conducted. Further large-scale studies and further examination are needed to develop the new strategy for MC.

## Conclusions

Genetic analysis of MC with infiltrative invasion, expansile invasion, and MBT revealed that ICIs might be an effective therapy for MC with infiltrative invasion. Further studies are needed to consider these relationships.

## Data Availability

All data generated or analysed during this study are included in this published article.

## Abbreviations

EOC: epithelial ovarian carcinoma
PARPi: poly ADP-ribose inhibitor
BRCA: breast cancer susceptibility gene
WHO: world health organization
MC: mucinous carcinoma
FIGO: International Federation of Gynecology and Obstetrics
MBT: mucinous borderline tumor
TP53: tumor suppressor protein p53
HER2: human epidermal growth factor receptor 2
DNA: deoxyribonucleic acid
FFPE: formalin-fixed paraffin-embedded
TMB: tumor mutation burden
Mut: mutations
Mb: Megabase
CREBBP: cyclic adenosine monophosphate response element binding protein binding protein
ICI: immune checkpoint inhibitor
